# Role of host factors in SARS-CoV-2 entry

**DOI:** 10.1016/j.jbc.2021.100847

**Published:** 2021-05-28

**Authors:** John P. Evans, Shan-Lu Liu

**Affiliations:** 1 Center for Retrovirus Research, The Ohio State University, Columbus, Ohio, USA; 2 Department of Veterinary Biosciences, The Ohio State University, Columbus, Ohio, USA; 3 Molecular, Cellular and Developmental Biology Program, The Ohio State University, Columbus, Ohio, USA; 4 Department of Microbial Infection and Immunity, The Ohio State University, Columbus, Ohio, USA; 5 Viruses and Emerging Pathogens Program, Infectious Diseases Institute, The Ohio State University, Columbus, Ohio, USA

**Keywords:** virus entry, RNA virus, endocytosis, membrane fusion, cathepsin B, TMPRSS2, ACE2, SARS-CoV-2, entry cofactor, virus receptor, ACE2, angiotensin converting enzyme 2, Cat B/L, cathepsin B/L, CD147, cluster of differentiation 147, Cyp A/B, cyclophilin A/B, HCoV, human coronavirus, HSPG, heparin sulfate proteoglycan, IFITM, interferon-induced transmembrane proteins, ISG, interferon-stimulated gene, Ly6E, lymphocyte antigen 6E, MERS-CoV, Middle East respiratory syndrome coronavirus, MHV, mouse hepatitis virus, NRP-1, neuropilin-1, PS, phosphatidylserine, RBD, receptor binding domain, SARS-CoV, severe acute respiratory syndrome coronavirus, SARS-CoV-2, severe acute respiratory syndrome coronavirus 2, TIM-1, T-cell immunoglobulin and mucin domain 1, TMPRSS2, transmembrane protease, serine 2

## Abstract

The zoonotic transmission of highly pathogenic coronaviruses into the human population is a pressing concern highlighted by the ongoing SARS-CoV-2 pandemic. Recent work has helped to illuminate much about the mechanisms of SARS-CoV-2 entry into the cell, which determines host- and tissue-specific tropism, pathogenicity, and zoonotic transmission. Here we discuss current findings on the factors governing SARS-CoV-2 entry. We first reviewed key features of the viral spike protein (S) mediating fusion of the viral envelope and host cell membrane through binding to the SARS-CoV-2 receptor, angiotensin-converting enzyme 2. We then examined the roles of host proteases including transmembrane protease serine 2 and cathepsins in processing S for virus entry and the impact of this processing on endosomal and plasma membrane virus entry routes. We further discussed recent work on several host cofactors that enhance SARS-CoV-2 entry including Neuropilin-1, CD147, phosphatidylserine receptors, heparan sulfate proteoglycans, sialic acids, and C-type lectins. Finally, we discussed two key host restriction factors, *i.e.*, interferon-induced transmembrane proteins and lymphocyte antigen 6 complex locus E, which can disrupt SARS-CoV-2 entry. The features of SARS-CoV-2 are presented in the context of other human coronaviruses, highlighting unique aspects. In addition, we identify the gaps in understanding of SARS-CoV-2 entry that will need to be addressed by future studies.

Coronaviruses represent a diverse family of enveloped, positive-sense, RNA viruses infecting birds and mammals, which have become of increasing concern following three recent zoonotic transmissions of highly pathogenic human coronaviruses including Middle East respiratory syndrome coronavirus (MERS-CoV), severe acute respiratory syndrome coronavirus (SARS-CoV), and severe acute respiratory syndrome coronavirus 2 (SARS-CoV-2). Divided into four genera, the alpha- and beta-coronaviruses mainly circulate in bat and rodent reservoirs, whereas the gamma- and delta-coronaviruses have birds as their main reservoir species (1, 2). Of these, the seven human coronaviruses (HCoV) are all from the alpha- and beta-coronavirus genera (1, 2). These include the four mildly pathogenic human coronaviruses that cause common colds, HCoV-229E, HCoV-OC43, HCoV-NL63, and HCoV-HKU1 (1, 2); the highly pathogenic human coronaviruses SARS-CoV and MERS-CoV, with case fatality rates near 10% and 30%, respectively (1); as well as the more moderately pathogenic yet highly transmissible human coronavirus SARS-CoV-2, which is causing the ongoing pandemic (1, 3) with a case fatality rate closer to 3% at the outset of the pandemic (1). The recent rate of zoonotic transmission of these more pathogenic coronaviruses is alarming with the introductions of SARS-CoV, MERS-CoV, and SARS-CoV-2 all having occurred within the past 2 decades (1, 3).

Since the identification of SARS-CoV-2 as the causative agent of COVID-19 in December of 2019 (4, 5, 6, 7), as of April 11, 2021, the World Health Organization reports cumulatively over 135 million documented COVID-19 cases resulting in 2.9 million deaths worldwide. In response, remarkable effort from the scientific community has sought to define the mechanisms of SARS-CoV-2 replication, transmission, and pathogenesis (8, 9, 10, 11). In particular, the mechanisms of SARS-CoV-2 entry were probed to better define the requirements for SARS-CoV-2 transmission and pathogenesis, as well as vaccine development. The SARS-CoV-2 receptor was quickly identified as angiotensin converting enzyme 2 (ACE2) (4, 7, 12), and subsequent studies have revealed other key determinants of SARS-CoV-2 entry. Factors influencing viral entry can serve as key determinants of virus host range, tissue tropism, and pathogenicity and may be targets for therapeutics and vaccine development. Here we review the recent findings related to SARS-CoV-2 entry and place them in the context of prior coronavirus research as well as identify areas requiring further investigation.

## Receptor binding: What is unique about SARS-CoV-2 spike?

The coronavirus spike (S) protein, one of four structural proteins, E, M, N, and S, mediates receptor binding and fusion of virus particles with target cells. The S protein is a typical type I fusion protein and functions as a trimer with each monomer divided into two subunits: S1, which mediates receptor binding, and S2, which contains the transmembrane domain and mediates fusion with the host cell membrane. Of interest, distinct from SARS-CoV yet similar to MERS-CoV, the SARS-CoV-2 S protein contains a furin proteolytic cleavage site at the S1/S2 junction, which aids in efficient viral entry, spread, and pathogenesis (13). The S proteins of various coronaviruses interact with a broad range of receptors including ACE2, dipeptidyl peptidase 4, aminopeptidase-N, and sialic acid moieties to facilitate the entry process. This diversity of receptor utilization may help explain the strong potential for zoonotic transmission of coronaviruses to humans.

Despite using the same receptor, ACE2, for binding and entry, the dynamics and receptor binding properties of SARS-CoV-2 S are somewhat different from that of SARS-CoV. Early observations indicated that the receptor-binding domain (RBD) of SARS-CoV-2 S exhibited higher affinity for ACE2 than the RBD of SARS-CoV S (11, 14, 15), and cryo-EM structures of SARS-CoV and SARS-CoV-2 RBD in complex with ACE2 demonstrated more extensive interactions between SARS-CoV-2 RBD and ACE2 (12, 14). However, subsequent reports have suggested that full-length SARS-CoV-2 S exhibits a similar or weaker affinity for ACE2 compared with SARS-CoV S (11, 16, 17). One possible explanation for this discrepancy is that the RBD for SARS-CoV-2 S may be less accessible to ACE2. Prior structural analyses of SARS-CoV and MERS-CoV identified an equilibrium in S proteins between an RBD-exposed conformation (open) and RBD-buried conformation (closed) (18, 19). More recently, cryo-EM-resolved SARS-CoV-2 S structures have detected a greater proportion of RBD in a closed conformation (12, 17, 20) compared with SARS-CoV or MERS-CoV (18, 19). This may indicate that SARS-CoV-2 S spends more time in the closed conformation than SARS-CoV S, potentially causing SARS-CoV-2 S to have a comparable affinity to ACE2 as SARS-CoV S despite the higher affinity for ACE2 of SARS-CoV-2 RBD. This may also imply an immune evasion strategy of SARS-CoV-2, where the RBD is hidden in a closed conformation to prevent the development of neutralizing antibody responses against the RBD of SARS-CoV-2 S. Further study of the triggers for a closed to open S conformational change are required to better understand how SARS-CoV-2 balances immune evasion strategies with efficient entry.

## Entry pathway and protease utilization: From plasma membrane to endosome

**Figure 1:**
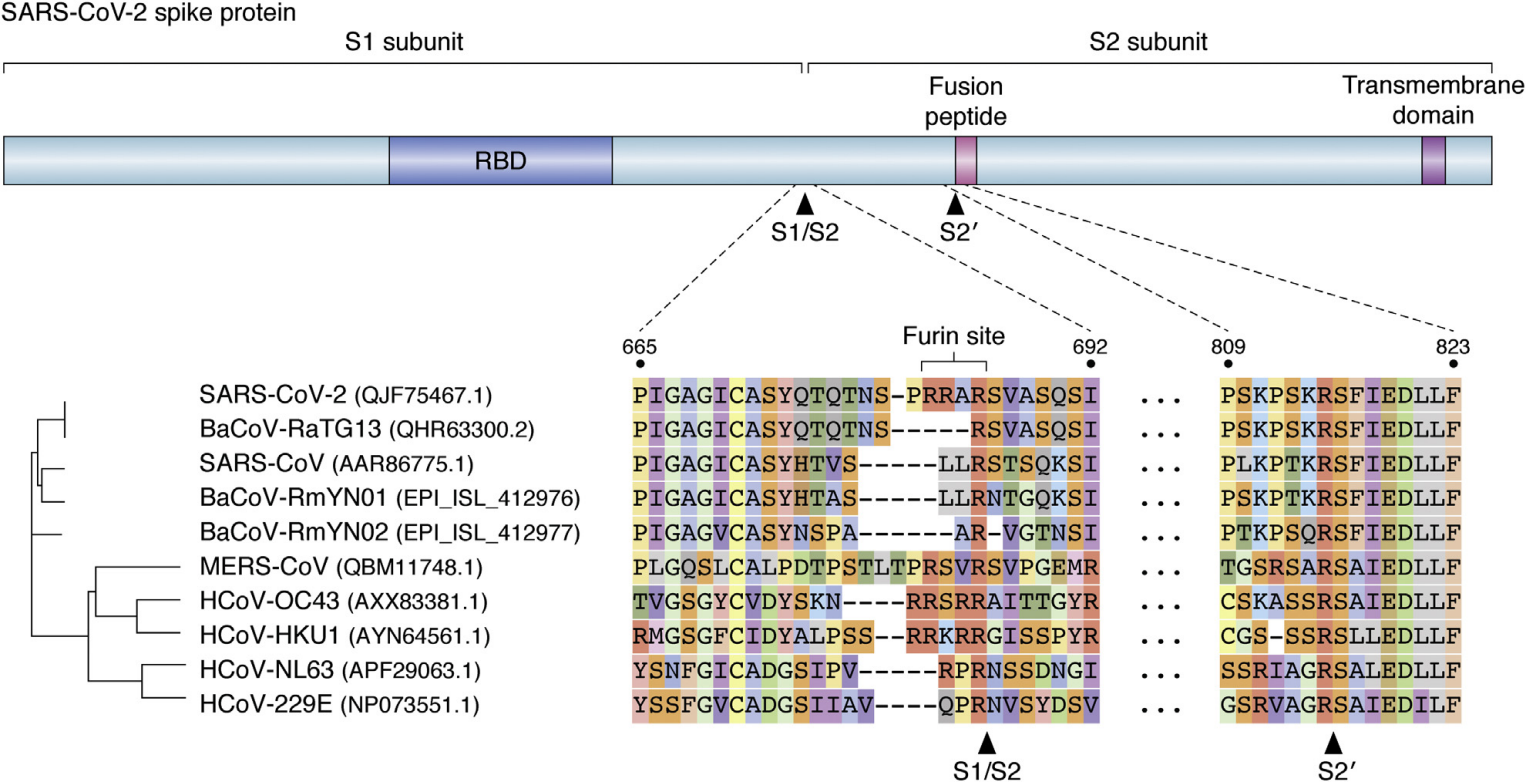
**SARS-CoV-2 Spike contains a furin cleavage motif at S1/S2 cleavage site.***Top*, schematic of the SARS-CoV-2 Spike protein with S1 subunit, S2 subunit, receptor binding domain (RBD), fusion peptide, transmembrane domain, S1/S2 cleavage site, and S2’ cleavage site indicated. *Bottom*, alignment of SARS-CoV-2 S1/S2 and S2’ cleavage sites with corresponding regions of S protein from related bat coronaviruses (BaCoV) and other human coronaviruses. The phylogenetic tree indicates the relatedness of full-length S proteins. The RXXR furin cleavage motif at the S1/S2 site is indicated for SARS-CoV-2 and is present in MERS-CoV, HCoV-OC43, and HCoV-HKU1. The site of cleavage is indicated with an *arrowhead*. Sequence IDs are indicated next to the virus names and correspond to NCBI accession numbers or GISAID accession numbers. Alignment and phylogenetic tree were produced using full-length S protein sequence alignment with ClustalOmega (130).

**Figure 2:**
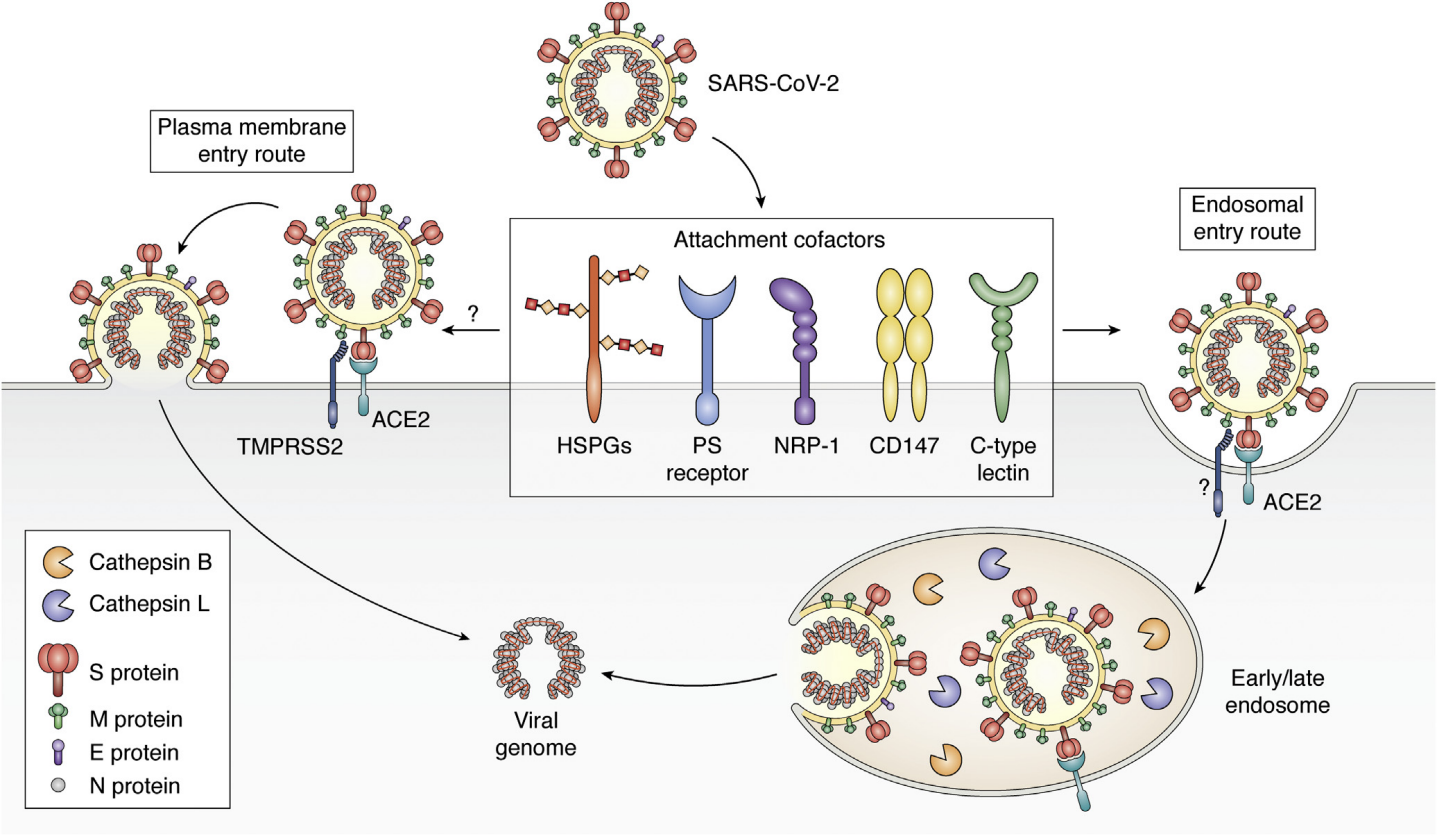
**SARS-CoV-2 attachment cofactors can enhance virus entry *via* the endosomal entry route and the plasma membrane entry route.** Binding of virions to the representative attachment cofactors can facilitate SARS-CoV-2 S binding to ACE2. Then subsequent cleavage by cell surface TMPRSS2 can lead to cell membrane fusion, or endocytosis of SARS-CoV-2 allows for cathepsin B/L processing of SARS-CoV-2 S and subsequent fusion both in an ACE2-dependent manner. Whether or not TMPRSS2 processing could influence endosomal entry is currently unknown.

As most class I viral fusion proteins, processing of the coronavirus S protein by host proteases is required for coronavirus entry. This priming process involves a cleavage at the S1/S2 boundary of the precursor S, a cleavage at the S2’ site of the S protein, or both, which results in a dissociation of the S1 subunit from S2 thus allowing S2 subunit–mediated membrane fusion between the viral envelope and the cell membrane (21) (Figs. 1 and 2). Previous reports have demonstrated that SARS-CoV can be primed at the S2’ site by cell membrane–associated transmembrane protease serine 2/4 (TMPRSS2/4) (22, 23), endosomal cathepsin B/L (CatB/L) (22, 24), and other trypsin-like proteases (22, 25). Similarly, recent evidence utilizing SARS-CoV-2 spike-pseudotyped viruses, or viral particles from commonly studied viruses bearing the SARS-CoV-2 S protein, has shown that SARS-CoV-2 can be activated *via* the same set of cell surface and endosomal proteases. Indeed, pharmacological inhibition of TMPRSS2 or CatB/L has been shown to reduce SARS-CoV-2 S-pseudotyped vesicular stomatitis virus or lentivirus entry (11, 26). Moreover, inhibition of TMPRSS2 with camostat mesylate is being pursued as a potential COVID-19 therapy (27). Noticeably, the furin cleavage site present at the SARS-CoV-2 S1/S2 boundary is not otherwise present in most lineage B beta-coronaviruses, including the closely related bat CoVs RaTG13, RmYN01, and RmYN02 as well as relatively divergent SARS-CoV (Fig. 1). However, similar furin cleavage sites do exist in the S proteins of MERS-CoV, HCoV-HKU1, and HCoV-OC43 and many other animal coronaviruses (Fig. 1) (13, 21). This furin cleavage site allows for proteolytic processing of SARS-CoV-2 S in the virus producer cell, typically in the *trans*-Golgi complex, rather than during entry into target cells. Mutation of the furin cleavage site drastically reduces the infectivity of SARS-CoV-2 S pseudotyped virus (11), and virus produced from cells treated with a furin inhibitor also demonstrate a greater sensitivity to TMPRSS2 or CatB/L inhibitors for infection in HeLa cells overexpressing ACE2 (11). This suggests that the presence of the furin cleavage site at the S1/S2 boundary in SARS-CoV-2 S likely reduces reliance on target cell proteases. This is unusual as cleavage at the S2’ site has been thought to be the major requirement for SARS-CoV entry (21). Of interest, infectious SARS-CoV-2 possessing a furin cleavage site deletion exhibits enhanced replication in Vero-E6 cells but reduced replication in the lung epithelia-derived Calu-3 cell line (28). This may indicate a need for the furin cleavage site for replication and pathogenesis in the lungs that is not necessarily maintained in all cell culture systems. It is thus not surprising that the furin cleavage site is deleted in cells lacking expression of TMPRSS2, such as Vero-E6 cells, when SARS-CoV-2 is serially passaged (29, 30). Further investigation is needed to determine the relative importance of these proteases and cleavage events in determining SARS-CoV-2 entry, pathogenesis, tropism, and host range.

The flexible protease usage of SARS-CoV-2 highlights the inherent plasticity of the virus in entry pathway usage (Fig. 2). SARS-CoV-2, SARS-CoV, and MERS-CoV are all capable of using both endosomal and plasma membrane entry routes (26, 31, 32). TMPRSS2 appears to be one of the major proteases for priming S for entry *via* the plasma membrane, whereas CatB/L performs the priming function during entry through the endosome. Supporting this notion, endosomal acidification inhibitors have been shown to efficiently block SARS-CoV and SARS-CoV-2 entry by preventing the activation of CatB/L (26, 31). However, low pH *per se* does not appear to act directly as a trigger for SARS-CoV-2 entry (20). It should be noted, however, that recent reports suggest that pH 5.5 to 6.0 can stabilize the SARS-CoV-2 S protein and cause a shift toward a more open conformation, potentially facilitating viral membrane fusion in the endosome (20). This stability and opening of the S protein at endosomal pH may suggest a preference for an endosomal entry route. However, cell culture studies have demonstrated that TMPRSS2 inhibitors more drastically reduce SARS-CoV-2 entry compared with CatB/L inhibitors in physiologically relevant cell types such as Calu-3 cells (26). In addition, it has been shown for MERS-CoV that furin cleavage in producer cells leads to a preference for subsequent processing by TMPRSS2 (32, 33). Prior reports suggest that other human coronaviruses, such as HCoV-OC43 and HCoV-HKU1, exhibit a preference for a cell membrane entry route and only acquire an ability to utilize CatB/L upon passaging in HBTE-ALI cell culture while accumulating a furin cleavage site mutation (34). This is noteworthy as the passaging of SARS-CoV-2 in Vero-E6 cells has led to the problematic accumulation of furin cleavage site deletions or mutations (29, 35, 36), perhaps suggesting a shift toward an endosomal entry route in Vero-E6 culture. Hence, the flexibility in protease usage and entry route appears to be a consistent strategy used by coronaviruses, despite in some cases acting in a cell type–dependent manner. More investigation is needed to determine the physiological relevance of each entry pathway to SARS-CoV-2 spread and pathogenesis *in vivo*.

## Cellular entry modulators: Receptor *versus* cofactor

Spike/receptor interactions and protease processing of the spike are not the only factors determining coronavirus entry. A number of cellular proteins, referred to as cofactors here, have been identified to enhance the attachment and entry of coronavirus particles into target cells. Such entry cofactors are sometimes referred to as viral receptors in the literature, which has caused much confusion. Although defining a viral receptor can be challenging, it should include at least a demonstration of the direct interaction with viral protein of interest *in vitro* and the induction of conformational changes in the viral protein (37). Caution should be exercised in distinguishing a *bona fide* receptor from a cellular cofactor. Currently, ACE2 is the only primary receptor identified for SARS-CoV-2, the highly related SARS-CoV, as well as distantly related HCoV-NL63. Here we outline important coronavirus entry cofactors and ongoing work to determine their role, if any, in SARS-CoV-2 entry (Table 1).

**Table 1:** Host factors affecting human coronavirus entry

Virus	Receptor	Cofactor	Restriction factor
SARS-CoV-2	ACE2 (4, 7, 12)	TMPRSS2 (11, 26)	IFITMs (103, 104, 105)
		CatB/L (11, 26)	
		NRP-1 (38, 39)	
		CD147 (51)	Ly6E (108, 109)
		Axl (57)	
		TIM-1^a^ (63)	
		HSPGs (73, 74, 75, 76)	
		C-type lectin (91, 93)	
SARS-CoV	ACE2 (115)	TMPRSS2 (22, 23)	IFITMs (100)
		CatB/L (22, 24)	
		CypA/B (41, 48, 49)	Ly6E (109)
		HSPGs (70)	
		C-type lectin (89, 90, 92)	
MERS-CoV	DPP4 (116)	TMPRSS2 (32, 33, 117)	IFITMs (99)
		CatB/L (118)	
		CypA/B (119)	Ly6E (108, 109)
		Sialic Acid (83, 84)	
HCoV-NL63	ACE2 (120)	CypA/B (47)	IFITMs (102)
		HSPGs (67)	Ly6E (108)
		C-type lectin (121)	
HCoV-OC43	9-O-Ac-Sia (122)	TMPRSS2 (34)	Ly6E (108, 109)
		CatB/L (34)	
		HSPGs (123)	
		HLA-1 (124)	
		IFITMs (101)	
HCoV-HKU1	9-O-Ac-Sia (78)	TMPRSS2 (34)	
		CatB/L (34)	
		HLA-C (125)	
HCoV-229E	hAPN (126)	TMPRSS2 (127)	IFITMs (102)
		Cat B/L (128)	
		CypA/B (46, 48)	Ly6E (109)
		C-type lectin (129)	

Abbreviations: 9-O-Ac-Sia, 9-O-acetylated sialic acid; DPP4, dipeptidyl peptidase 4; hAPN, human aminopeptidase-N; TMPRSS2, Transmembrane protease, serine 2.

a Designation is based solely on data that has not yet undergone peer review.

Neuropilin-1 (NRP-1) has recently been identified as an entry cofactor for SARS-CoV-2 (38, 39). It is intriguing that NRP-1 was shown to bind furin cleavage products containing an R/KXXR/K motif (40). Given the presence of such a furin cleavage site in the S of SARS-CoV-2, two groups recently investigated the role of NRP-1 in SARS-CoV-2 S-mediated entry (38, 39). NRP-1 knockdown in Caco-2 cells, or knockout in HeLa cells stably expressing ACE2, reduces infectious SARS-CoV-2 replication (39). In addition, overexpression of NRP-1 in Caco-2 cells, which endogenously express ACE2, enhanced SARS-CoV-2 S pseudotyped virus entry (38), indicating a role for NRP-1 in SARS-CoV-2 entry. However, overexpression of NRP-1 in HEK 293T cells only enhances SARS-CoV-2 pseudotyped virus infection when both ACE2 and TMPRSS2 are coexpressed (38). Hence, it appears that NRP-1 does not function as a *bona fide* receptor for SARS-CoV-2 as entry still requires ACE2, indicating that NRP-1 is likely serving as an entry cofactor. Of note, NRP-1 seems to directly bind SARS-CoV-2 S as shown by coimmunoprecipitation and isothermal titration calorimetry (39), and this interaction is ablated for SARS-CoV-2 S with a furin cleavage site mutation (39). Furthermore, SARS-CoV-2 S pseudotyped virus entry is not perturbed by NRP-1 antibody blockade for S containing a furin cleavage site mutation (38). This would indicate that NRP-1 enhancement of SARS-CoV-2 entry is dependent on furin cleavage of the S protein. Indeed, expression of NRP-1 in the olfactory epithelium and airway (38) appears to facilitate SARS-CoV-2 entry despite the modest ACE2 expression seen in the human airway. In addition, silver nanoparticles coated with furin–cleaved SARS-CoV-2 S mimetic peptides were also more efficiently taken up by the olfactory epithelium and central nervous system of mice than nanoparticles coated with uncleaved S mimetic peptides (38). Whether or not NRP-1 functions in other tissues and is related to COVID-19 pathogenesis needs to be determined.

Cluster of differentiation 147 (CD147) has been shown to enhance the entry of several enveloped viruses (41, 42, 43, 44). This is due to binding of CD147 to virion-associated cyclophilin A or B (CypA/B) (41, 42, 45). In fact, the replication of HCoV-229E, HCoV-NL63, mouse hepatits virus (MHV), transmissible gastroenteritis virus, infectious bronchitis virus, feline coronavirus, and SARS-CoV has been found to be dependent on CypA/B and blocked by the CypA/B inhibitor cyclosporin A (46, 47, 48, 49, 50). Specifically, SARS-CoV N protein has been shown to interact with CypA and enhance SARS-CoV entry *via* CD147/CypA interactions on target cells (41). In addition, CD147 blockade was shown to reduce SARS-CoV-2 replication in Vero-E6 cells and direct interaction was seen between recombinant SARS-CoV-2 S and CD147 (51). However, it remains to be determined if CypA/B or SARS-CoV-2 N plays a role in entry enhancement by CD147. Although CD147 has been claimed as a *bona fide* receptor for SARS-CoV-2 (51, 52), current data seem to support the notion that CD147 acts as an attachment cofactor to facilitate SARS-CoV-2 entry. It is interesting that disruption of CD147 and CypA/B enhancement of SARS-CoV-2 entry has been suggested as a viable therapeutic strategy for the treatment of COVID-19 (52, 53, 54); however, the mechanism of CD147 enhancement of SARS-CoV-2 entry as well as its possible role in SARS-CoV-2 pathogenesis is currently unclear and requires investigation.

Phosphatidylserine (PS) receptors are another key set of viral entry cofactors. These PS receptors largely act by binding to PS, a modified membrane lipid, that is incorporated into enveloped or nonenveloped virus particles to enhance their attachment to target cells (37, 55). This process of PS-mediated enhancement of virus attachment is known as apoptotic mimicry and has been described for numerous enveloped viruses (55, 56). The PS receptor Axl, a member of the TAM family of PS receptors, was recently demonstrated to enhance the entry of infectious SARS-CoV-2 and SARS-CoV-2 pseudotyped lentivirus (57). Such Axl-dependent entry was also shown to occur in ACE2 knockout H1299 cells, potentially indicating an ACE2-independent entry route that relies on Axl (57). Another relevant PS receptor is the T-cell immunoglobulin and mucin domain type-1 (TIM-1) protein, which has been shown to enhance the entry of several enveloped viruses (37, 58, 59), and is a major cofactor facilitating ebolavirus endosomal entry (60, 61). Of note, TIM-1 failed to enhance the entry of SARS-CoV pseudotyped virus, but this was only tested in refractory cell lines (62). As TIM-1 is not expected to act as a *bona fide* receptor (37), it is unsurprising that TIM-1 did not render refractory cell lines permissible to SARS-CoV pseudotyped virus entry, and TIM-1 may still have an effect on SARS-CoV entry in higher ACE2-expressing cell lines. A recent report has found that TIM-1 does enhance the uptake of SARS-CoV-2 S incorporating lipid nanoparticles and this enhancement could be blocked by TIM-1 antibody blockade (63). However, these nanoparticles lack PS, raising the question of what might be mediating this uptake. Microscale thermophoresis data suggested that TIM-1 directly interacts with SARS-CoV-2 S (63), providing one possible explanation. Further investigation is needed to determine the role of TIM-1 on SARS-CoV-2 entry, the importance of the PS-binding activity of TIM-1, as well as the effect of TIM-1 on different SARS-CoV-2 entry routes. Given that TIM-1 has been shown to block the release of HIV-1 by trapping PS-incorporating virions on the virus producer cell (37, 64, 65), it would be interesting to investigate the role of TIM-1, if any, on SARS-CoV-2 release.

Another important factor promoting viral entry is heparan sulfate proteoglycans (HSPGs). Similar to PS receptors, this class of extracellular matrix glycosaminoglycans can enhance the entry of many viruses (66). Previous studies have demonstrated that the introduction of cell-free HSPG reduced HCoV-NL63 attachment and replication (67). In addition, treatment of MHV, a model coronavirus, with heparin, which is closely related to HSPG (68), was shown to reduce virus uptake and replication, similar to treatment of target cells with heprinase (69). Treatment with heprinase or exogenous heparin also reduced SARS-CoV pseudotyped virus infection and blocked binding of soluble SARS-CoV S to target cells (70). All these results suggest that the mechanism of HSPG-mediated enhancement of coronavirus infection may be the result of direct interaction between HSPG and Spike. For SARS-CoV-2, recent reports based on surface plasmon resonance, circular dicromism, ELISA, sepharose pulldown, and microarray data have established that SARS-CoV-2 S interacts with HSPG and heparin and that binding heparin may induce a conformational change in S (71, 72, 73, 74, 75). Heparin has also been shown to block the entry of SARS-CoV-2 pseudotyped vesicular stomatitis virus (73) and murine leukemia virus (74) as well as infectious SARS-CoV-2 (73, 76). Other sulfated polysaccharides have also been shown to disrupt infection by SARS-CoV-2 pseudotyped virus and infectious SARS-CoV-2 (76, 77). Furthermore, the development of inhibitors to block the interaction of SARS-CoV-2 S with HSPGs has been proposed as a potential therapeutic strategy for combating COVID-19 (74, 76). Collectively, this evidence suggests that HSPG may serve as an entry cofactor for SARS-CoV-2; however, the mechanism of action as well as its role in virus spread and pathogenesis remains unclear and warrants further investigation.

When not serving as the primary coronavirus receptor as for HCoV-HKU1, HCoV-OC43, and Bovine-CoV (78, 79, 80), sialic acids can still act as important coronavirus entry cofactors. These abundant cell-surface glycans serve as receptors and attachment cofactors for a wide range of viruses, including influenza (81, 82). The S protein of MERS-CoV has been shown to hemagglutinate red blood cells (83), which are highly decorated with sialic acid moieties, and MERS-CoV S-coated nanoparticles can bind to sialic acid moieties (83). In addition, the structure of MERS-CoV S-interacting with the sialic acid Neu5Ac has been solved (84). Of importance, neuraminidase digestion of sialic acid moieties on Calu-3 cells reduces MERS-CoV replication (83). However, it has recently been reported that SARS-CoV-2 S failed to bind any residues in a sialic acid microarray (85), indicating that a more sensitive assay may be required to detect SARS-CoV-2 S binding of sialic acids.

The C-type lectins, L-SIGN and DC-SIGN, also play an important role in enhancing viral entry (86, 87, 88). The S protein of SARS-CoV has been shown to bind DC-SIGN (89), and DC-SIGN can enhance the entry of SARS-CoV S pseudotyped lentivirus particles (90, 91) with specific S glycosylation site mutants failing to facilitate SARS-CoV S pseudotyped virus entry into HeLa cells overexpressing L-SIGN or DC-SIGN (92). Similarly, it has been recently reported that SARS-CoV-2 S can bind both DC-SIGN and L-SIGN and exogenous expression of either DC-SIGN or L-SIGN can enhance SARS-CoV-2 pseudotyped virus entry into HEK 293T cells (93). Although these cellular factors are claimed as *bone fide* receptors for SARS-CoV-2 (93, 94), available data seem to suggest a role as attachment cofactors. Thus, probing the effect of L-SIGN/DC-SIGN on SARS-CoV-2 entry of ACE2 knockout cells should help clarify their role.

A better understanding of the cellular cofactors enhancing SARS-CoV-2 entry, as discussed above, will further elucidate SARS-CoV-2 tissue and host tropism. In addition, such an understanding may allow for the development of therapeutics disrupting the entry phase of the SARS-CoV-2 lifecycle. However, all these entry cofactors require further investigation to clarify the mechanisms for enhancement of SARS-CoV-2 entry and to distinguish entry cofactors from *bona fide* receptors, especially in physiologically relevant systems.

## Entry restriction factors: Another side of the coin

Host restriction factors are another set of important modulators that can act on different steps of viral replication (95, 96). Although they are typically induced by type I interferon and often antagonized by a viral factor to allow evasion of host restriction, some exceptions do exist, such as serine incorporator (SERINC) proteins, which impair HIV infectivity (95, 96). Some of these restriction factors can inhibit viral entry and could also have additional or even opposite functions on other steps of viral replication, such as assembly and release. Here we focus on recent investigations into the roles of interferon-induced transmembrane (IFITM) proteins and lymphocyte antigen 6 complex locus E (Ly6E) in restriction of SARS-CoV-2 entry (Table 1).

The IFITM proteins are a class of interferon-stimulated genes (ISGs) that block viral membrane fusion with target cells (97, 98). These restriction factors have a complicated role in coronavirus replication. It has been demonstrated that knockdown of IFITM3 enhanced entry of HCoV-NL63, HCoV-229E, SARS-CoV, and MERS-CoV S bearing pseudotyped virus (99, 100). In addition, overexpression of IFITM1 to 3 inhibited infectious SARS-CoV replication and pseudotyped virus entry (100). However, IFITM3 has been shown to enhance the entry of HCoV-OC43 (101), and disruption of the N-terminal domain of IFITM3, specifically at tyrosine 20 (Y20) in an endosomal sorting motif, has been shown to allow IFITM3 to enhance the entry of SARS-CoV and MERS-CoV (102). These dual roles of IFITMs in coronavirus entry are reflected in recent SARS-CoV-2 studies. For instance, knockdown of IFITMs in Calu-3 or Caco-2 cells enhanced entry, whereas overexpression of IFITMs inhibited entry in HEK 293T cells overexpressing ACE2 (HEK 293T-ACE2) (103, 104). Similarly, overexpression of IFITMs inhibited infectious SARS-CoV-2 replication in HEK 293T-ACE2 (103, 104). Overexpression of IFITMs, especially IFITM1, inhibits syncytia formation of cells overexpressing ACE2 with cells expressing SARS-CoV-2 S, and expression of TMPRSS2 can counteract this inhibitory effect (105). However, knockdown of IFITMs, especially IFITM2, in the more biologically relevant Calu-3 cell line decreased infectious SARS-CoV-2 replication (103). In addition, introduction of a Y20A mutation in IFITM3 converted IFITM3 into an enhancer of infectious SARS-CoV-2 replication in HEK 293T-ACE2 cells (104). It is also interesting to note that overexpression of TMPRSS2 allowed for IFITM3 restriction of SARS-CoV-2 entry to be overcome (104). Our unpublished data showed that the effect of IFITMs on viral entry is cell type dependent and may be related to the level and stability of ACE2 expression on the plasma membrane (Qu *et al.*, unpublished data). Given that mutation of an endosomal sorting motif in IFITM3 ablates its restriction of SARS-CoV-2 and that expression of TMPRSS2, presumed to enhance SARS-CoV-2 entry at the cell membrane, can overcome IFITM3 restriction, these results indicate that IFITM3 likely restricts SARS-CoV-2 entry primarily in the endosome, which could be modulated by other cellular cofactors. The mechanism for IFITM-mediated differential effects on SARS-CoV-2 entry and replication in physiologically relevant cells requires further investigation.

Ly6/uPAR family member lymphocyte antigen 6 complex locus E (Ly6E) is an interferon-inducible ISG that has previously been shown to enhance viral entry for several enveloped viruses, including HIV (106, 107). However, for HCoV-OC43, overexpression of Ly6E was shown to inhibit virus entry and infection in HEK293 and A549 cells, whereas knockdown of Ly6E enhanced viral infection in HepG2 cells (108). In addition, overexpression of Ly6E was shown to inhibit entry of lentiviral pseudotyped virus bearing S from HCoV-OC43, HCoV-229E, HCoV-NL63, MERS-CoV, or SARS-CoV-2 (108, 109) and inhibited infection of their replication-competent viruses (109). Finally, Ly6E knockout mice exhibited more severe MHV infection (109). Although these results indicate Ly6E is a broadly acting restriction factor for coronaviruses that acts on virus entry, the effect can be cell type dependent, as we have shown for HIV (107, 110).

Owing to space limitation, we only highlight here the IFITM and Ly6E family proteins, with published effects on SARS-CoV-2, to demonstrate the role of ISGs in limiting coronavirus infection and pathogenesis; there are additional host restriction factors, including TRIM56 and tetherin (111, 112), that critically regulate different steps of replication for other coronaviruses. Of importance, viral proteins or antagonists have been reported to counteract some of these host restriction factors, including for SARS-CoV (112). It is this mode of virus–host evolutionary arms race that drives their coadaptation, which accounts in part for the emergence of new variants or species that spills over from animals to humans resulting in endemic or pandemic of infectious diseases such as COVID-19 (96, 113, 114).

## Concluding remarks

Coronavirus entry is characterized by a plasticity of entry routes regulated by receptor utilization, cofactor modulation, protease cleavage, as well as host restriction factors. Although much progress has been made in each of these aspects, there is a need for more investigation of their mechanisms of action, especially with respect to SARS-CoV-2 entry and how these factors interact with each other and ultimately dictate the entry pathways, *i.e.*, endosomal or plasma membrane routes. Understanding the complex mechanisms of SARS-CoV-2 entry may inform strategies for the design of effective therapeutics to disrupt the virus life cycle. More broadly and also significantly, a better grasp of coronavirus entry will help inform our understanding of the zoonotic transmission, spillover, and pandemic potential of future emerging coronaviruses.

## Conflict of interest

The authors declare no conflicts of interest with the contents of this article.
